# Culture and PCR based detection of bacteria causing urinary tract infection in urine specimen

**DOI:** 10.12669/pjms.36.3.1577

**Published:** 2020

**Authors:** Ghulam Sarwar Pirkani, Mohammad Arif Awan, Ferhat Abbas, Mohammad Din

**Affiliations:** 1 Ghulam Sarwar Pirkani, M.Phil. Pathology Department, Bolan University of Medical and Health Sciences, Quetta, Pakistan; 2 Mohammad Arif Awan, PhD. Center for Advanced Studies in Vaccinology and Biotechnology, University of Balochistan, Quetta, Pakistan; 3 Ferhat Abbas, PhD. Center for Advanced Studies in Vaccinology and Biotechnology, University of Balochistan, Quetta, Pakistan; 4 Mohammad Din, M.Phil. Pathology Department, Bolan University of Medical and Health Sciences, Quetta, Pakistan

**Keywords:** Antimicrobial susceptibility, *bla*NDM1, Disc diffusion, Multi drug resistance, Urinary Tract Infection (UTI)

## Abstract

**Objectives:**

Urinary tract infections are the second most common bacterial infections occurring at all ages and both sexes. The increasing rate of antibiotic resistance is a global concern. The use of routinely used antibiotics is resulting in treatment failure. The objective of this study was to diagnose the urinary tract infections by routine culture sensitivity test and by molecular methods.

**Methods:**

This study was conducted in Microbiology laboratory, Bolan Medical Complex Hospital, Quetta, from July 1^st^ to 31^st^ March 2019. Isolates were identified biochemically by API20E & API20NE. Antibiogram was performed using disc diffusion Kirby Bauer technique. The 16S rDNA gene approach was used for molecular identification of bacterial isolates. The presence of the *bla*_NDM-1_ gene was identified by polymerase chain reaction (PCR).

**Results:**

We isolated 146 bacterial isolates namely *Escherichia coli* (n=99) 67.80%, *Klebsiella* *pneumoniae* (n=33) 22.60%, *Pseudomonas aeruginosa* (n=11) 7.53% and *Proteus mirabilis* (n=3) *2.05%* from 2032 urine samples. The resistance pattern was dominated by Multi Drug Resistance (MDR). Remarkably, four isolates of *Escherichia coli* (n=3) and *Klebsiella* *pneumoniae* (n=1) were displaying resistance against a range of antibiotics used in the study, including carbapenems but sensitive to tigecycline and polymyxins only, suggesting extensive drug resistance having *bla* NDM-1 gene.

**Conclusion:**

This is the first report on direct molecular detection of bacterial pathogens from urinary tract infected patients in Balochistan. The presence of *bla*_NDM-1_ in different bacterial species and their extensive drug resistance pattern poses a significant clinical threat. Molecular detection of bacteria and resistant gene may reduce the diagnostic time of patients.

## INTRODUCTION

Urinary tract infection (UTI) results from the presence and multiplication of microorganisms, in one or more structures of the urinary tract. Ninety-five percent UTI cases are due to bacteria.^1^ Globally, about 150 million people are prone to urinary tract infections every year.^2^ With bacterial infections, urinary tract infection is the second most common type.^3^ The most common bacteria causing UTI, are *Escherichia coli*, *Klebsiella* *pneumoniae*. *Pseudomonas aeruginosa, Proteus* spp *, Staphylococcus saprophyticus* and *Enterococcus* spp.^4^ UTI is commonly diagnosed by urine analysis.^5^ The presence of pus cells in the urine recommends the culture and sensitivity test, which takes 48-72 hour awaiting the final report. Urine examination and confirmed by isolation of uropathogen in urine culture while bacteria present >1,000 cfu/ml of urine is a standard threshold. The above mentioned diagnostic procedures are time-consuming, and take at least three days. A rapid, definitive urine test capable of detecting bacteria would be beneficial in ensuring timely treatment, and in eliminating empirical treatment. Recently, many PCR-based gene tests have been developed for bacterial identification in other body infections. 16S rRNA gene is a well-characterized bacterial-specific bio signature used to detect and identify bacteria.^6^ While it is feasible to extract 16S rDNA from various infected bodily fluids, including urine as it can be obtained in a non-invasive manner.^7^

The present study, entail the analysis of urine of outdoor and admitted patient’s. The urine samples having leukocytes were subjected to routine culture sensitivity and PCR test. The detected bacteria by culture method and molecular method were analyzed. From resistant strains of isolated bacteria resistant gene have also been detected. The time taken by molecular method of detection of resistant gene is generally 3 to 4 hours.^8^ The goal of the study was to give the patients timely molecular based diagnosis and early relief to the affected individuals by using antibiotic of choice specially in case of resistant strains.

## METHODS

Two thousand and thirty two (n=2032) urine samples were collected aseptically in commercially available sterilized wide mouth containers from in and outdoor patients of tertiary care hospitals in Quetta. Demographic data was obtained with the consent of the patients. Samples were immediately sent after collection to the Microbiology laboratory of Bolan Medical Complex hospital, Quetta from July 1st to 31st March 2019. The study was approved by the Institutions Ethical Committee of BMC Hospital (No. E.C.4- 8/2017 dated May 24, 2017).

### Urine detail report

All the samples were screened biochemically by commercially available urine strips (Mission®, Accon laboratories, Inc.1025. Mesa Rim Road. San Diego, CA. USA) for protein, sugar and nitrite followed by microscopy of cells (Accon laboratories, Inc.1025. Mesa Rim Road. San Diego, CA. USA). Urine samples with leukocytes more than 10/ HPF were selected for further Studies.^9^ The selected urine samples were divided into two portions, one for culture and other for the PCR amplification.

### Bacterial isolation and Identification

Classical bacteriological procedures were used for bacterial isolation from selected urine samples. Each sample was mixed well and inoculated on Cystine Lactose Electrolyte Deficient (CLED) agar plates (Oxoid, United Kingdom) using a 5 mm diameter calibrated wire loop followed by incubation aerobically at 37°C for 24 hrs. Plates were observed for bacterial growth and the isolated colonies were further triple cloned. Bacterial isolates were identified by analytical profile index (API), API 20E and API 20NE system (bioMerieux, France) according to the manufacturer’s directions (Analytical Profile Index API).^10^ (http://www.biomerieux-usa.com/clinical/api). Bacterial genomic DNA was extracted using, Thermo Scientific Genomic Purification Kit, Lithuania, following the manufacturer’s instructions. 16S rDNA gene was amplified using universal primers, 27F-5’- AGA GTT TGA TCC TGG CTC AG -3’ and RD1-5’- AAG GAG GTG ATC CAG CC -3’ for the amplification of an internal fragment of 1500 bp. Applied Biosystem, USA thermocycler was used with Initial denaturation temperature, 95°C for two minutes, followed by 35 cycles of 30 seconds at 95°C, 30 seconds at 55°C and two minutes at 72°C. A final extension was carried at 72°C for 10 minutes.^11^. Sequencing of the PCR product of 16S rDNA genes of the representative samples was carried out commercially through Macrogen, South Korea. Sequences were aligned using Basic Local Alignment Search Tool (BLAST). https://blast.ncbi.nlm.nih.gov/Blast.cgi?PROGRAM=blastn&PAGE_TYPE=BlastSearch&LINK_LOC=blasthome.

### Antimicrobial Susceptibility

The standardized antibacterial sensitivity test was performed on Mueller-Hinton agar plates using disc diffusion Kirby Bauer technique with 0.5 McFarland turbidity standard methods and results were interpreted according to CLSI 2014 (CLSI. M100–S124.; 2014).^12^

### blaNDM-1 gene detection

Plasmid DNA was extracted for selected phenotypically carbapenem resistant isolates using GeneJET Plasmid Miniprep Kit by Thermo Fisher Scientific Lithuania, according to the manufacturer’s instructions. The blaNDM-1 gene was amplified by polymerase chain reaction (PCR) using primers; F-5’- GGG CAG TCG CTT CCA ACG GT-3’ and R-5’- GTA GTG CTC AGT GTC GGC AT -3’. Conditions for PCR were set to; initial denaturation at 95°C for five minutes, followed 30 Cycles of 95°C for 40 seconds, 58°C for 30 seconds, 72°C for 30 seconds with Final extension 72°C for 5 minutes.^13^ After amplification, DNA was loaded in 2% agarose gel and connected to the electrophoresis device Wealtec ELITE-300 (S.# E3W0578 UAS) by setting voltage 120 Amp, and 400 mA for 30 minutes. Electrophoresis gel was transferred to the gel documentation system Wealtec (USA) Dolphin-view S # WDV 50710004 for reading Sequencing of the PCR product of *bla* NDM-1 genes of the representative samples was carried out commercially through Macrogen, South Korea. Sequences were aligned using Basic Local Alignment Search Tool.^14^

## RESULTS

One hundred and seventeen (n=146) urine samples out of 2032, taken from both male and female patients categorized age wise and on the basis of pus cells were identified based on cultural, morphological and biochemical characterization, and API system (Table-I). Five urine specimen having leukocytes more than 20 per HPF had no growth on CLED agar. Two of them were found to be due to *Mycobacterium tuberculosis* infection on further investigation. Three patients were taking injectable antibiotics, have shown no growth on culture and no resistant gene on molecular analysis. Out of 146 bacterial isolates, the bacteria isolated were, *Escherichia coli*, *Klebsiella pneumoniae, Pseudomonas aeruginosa* and *Proteus mirabilis* were identified respectively (Table-II).

**Table-I:** Patients and their categorical data.

Total No.	OPD n (%)	Indoor n (%)	Male n (%)	Female n (%)	Age Categories/years			Pus Cells /HPF		
146	84 (57.53)	62 (42.46)	64 (43.83)	82 (56.16)	1-25	26-50	>50	11-30	30-50	>50
					64	58	24	63	57	26

**Table-II:** Number and percentage of isolated bacterial pathogens.

Name of organisms	Number (n)	Percentage
Escherichia coli	99	67.80
Klebsiella pnuemoniae	33	22.60
Pseudomonas aeruginosa	11	7.53
Proteus mirabilis	3	2.05

Antimicrobial sensitivity against a range of antibiotics, including those used in daily clinical practice and many broad spectrum are shown in (Table-III). All isolates belonging to four different spp, of *Escherichia coli*, *Klebsiella pneumoniae, Pseudomonas aeruginosa* and *Proteus mirabilis* were found, showing increased resistance against many broad spectrum antimicrobials. It is noted that uropathogens are becoming resistant to routinely prescribed oral antibiotics except nitrofurantoin. cephalosporin and quinolone drugs also gaining resistance.

**Table-III:** Resistance pattern of isolated bacteria from UTI cases.

Names of Pathogens	Resistant markers (Disc diffusion method)							

	F n (%)	PIP n (%)	NA n (%)	FOS n (%)	IPM n (%)	AK n (%)	CIP n (%)	CAZ n (%)
Escherichia coli (n=99)	7(7.07)	80(80.80)	90(90.90)	8(8.08)	3(3.03)	9(9.09)	74(74.74)	57 (57.57)
K.pneumoniae (n=33)	4(12.12),	30(90.90)	31(93.93)	5(15.15)	1(3.03)	1 (3.03)	7(21.21)	5(15.15)
P. aeruginosa (n=11)	Na	11(100)	11(100)	6 (54.54)	0	1 (9.09)	3(37.5)	3(27.27)
Proteus mirabilis (n=3)	Na	3(100)	3(100)	0(0)	0(0)	0(0)	0(0)	0(0)

Key: F: Nitrofurantoin. NA: Nalidaxic Acid. FOS: Fosfomycin. IPM: Imepenem. AK:Amikacin. CIP: Ciprofloxacin. OFX: Ofloxacin. CAZ: Ceftazedime. CTX: Cefatoxime. CRO: Ceftriaxone. Na: Not applicable.

**Fig.1:**
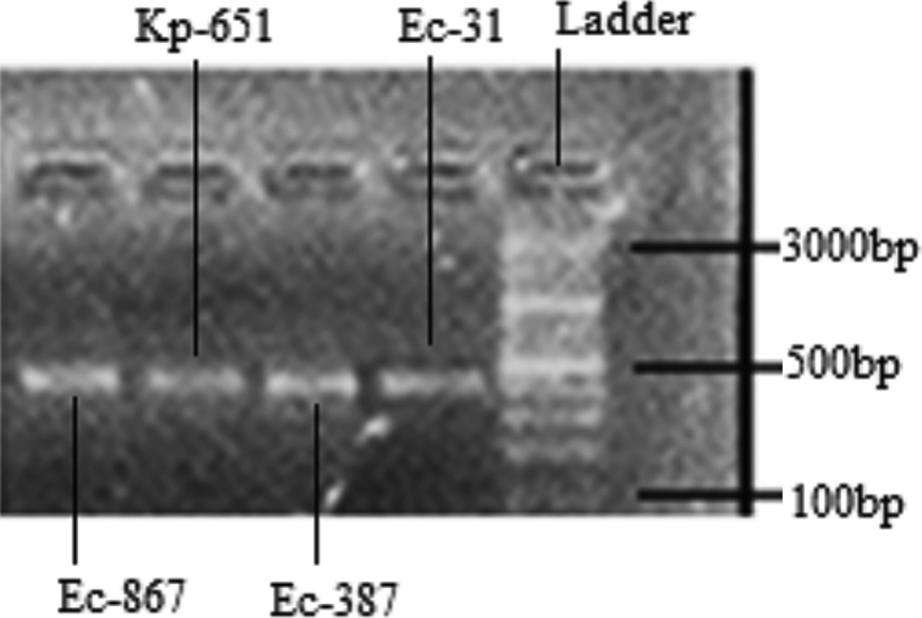
blaNDM-1 bands on 475bp position.

Four carbapenem resistant bacterial isolates of two different spp. namely *Escherichia coli* (Ec-31, Ec-387 & Ec-867 and *Klebsiella* *pneumoniae* (Kp-651) were positive for blaNDM-1 gene showing bands on 475bp position (Fig.1), *Escherichia coli* and *Klebsiella* *pneumoniae* harboring blaNDM-1 were resistant to all antibacterials including imepenem, meropenem and ertapenem while susceptible to polymaxin-B, tygacycline and colistin. (Table-IV).

**Table-IV:** Susceptibility pattern of *bla*NDM-1 positive isolates.

Isolate	IPM	MEM	ETP	CS	TGC	PB
Ec- 31	R	R	R	S	S	S
Ec- 387	R	R	R	S	S	S
Kp-651	R	R	R	S	S	S
Ec-867	R	R	R	S	S	S

Key:- Imipenem (IPM), Meropenem (MEM), Ertapenem (ETP), Colistin (CT), Tigecyclin (TGC), Polymyxin-B (PB).

## DISSCUSSION

The previous studies showed, urinary tract infection as the most common bacterial infections, prevalent in the hospitals and in the community.^15^ *Escherichia coli* were responsible for the majority of the infections.^16^

We have isolated bacterial pathogens of four different spp., *Escherichia coli, Klebsiella pneumoniae*, *Pseudomonas aeruginosa, and Proteus mirabilis*. In AKU Karachi urine samples were having *E. coli* (40%), *Pseudomonas* sp. (*16%), Klebsiella* sp. (11%), *Proteus* sp. (13%).^17^ In Gilgit Baltistan 47.7%, were *E. coli*, 41% *Klebsiella pneumonia* and 13.7%, *Enterococci* sp. In one of the recent studies from the USA, most common uropathogens remain *Escherichia coli* accounting about 70% of total cases followed by *Proteus mirabilis*, *Klebsiella* and *Enterobacter*.^18^ The *E. coli* and *Klebsiella* strains are becoming resistant to commonly used antibiotics. In the present study the most prevalent organism was *E. coli* (67.80%). Furthermore, we found that UTI is more common in females (56.16%), which is also in agreement with studies in the USA and in Pakistan.

Worldwide, there is gradual increase in antimicrobial resistance among uropathogens, in one of the study conducted in 2008 had found that *E. coli* was resistant against ampicillin cotrimoxazole, ciprofloxacin, gentamicin, nitrofurantoin and amikacin (92%), (80%), (62%), (47%), (20%) and (4%) respectively.^19^ Whereas, in our study resistance percentage against ciprofloxacin was 76% and Amikacin 9%, which is alarming in the region. In another study *E.coli* isolates were found susceptible to carbapenems (100%), amikacin (98.1%), cephalosporins, (96.2%) and piperacillin–tazobactam (88.5%), whereas fluoroquinolones were found highly potent against *E.coli*, but rate of high resistance to ciprofloxacin has also been observed.^20^ The *E.coli* and *Klebsiella* strains are becoming resistant against commonly used antibiotics^21^, and drugs used for prophylactic use.^22^ We found 3.03% and 9.09% resistance against imipenem and amikacin respectively in *E. coli* and *Klebsiella* strains which is an evidence of increasing resistance.

The rise in drug resistance is alarming, especially as new resistant gene NDM-1 has been discovered in *E. coli*, and *Klebsiella.^23^* From urine samples seven carbapenem-resistant NDM-1-positive *Klebsiella pneumoniae* isolates were recovered, from patients in different wards at a referral and tertiary care centers in Nairobi. All isolates were positive for blaNDM-1 carbapenemase gene.^24^ Molecular detection of bacteria is becoming a common mode of diagnosis, which not only reduces the diagnostic time but also helps in detection of resistance genes. In UK the *E. coli* was causing infections having extended-spectrum beta-lactamase (ESBL) which was confirmed in Health Protection Agency (HPA) report. These were predominantly of the type CTX-M-15. Most strains were resistant to beta-lactams and other classes of antibiotics and, in some cases, only carbapenem and aminoglycosides were susceptible.^25^ In the present study, we have isolated four blaNDM-1 positive isolates of *E*. *coli* and *Klebsiella*. The presence of NDM-1 in diverse microbial species and increasing antimicrobial resistance in urinary tract infections imposes precise and early detection, particularly in view of the limited treatment options available and where irrational use of antimicrobial in the region is a common practice. Moreover, it is also proved that molecular diagnosis is more reliable and less time consuming as compared to the traditional culture and sensitivity which is more time consuming in favor of the patients suffering from UTI,s.

## CONCLUSIONS

In brief *E.coli* is observed the most common bacteria causing urinary tract infection in all ages and both sexes but female patients suffer more than the male patients due to poor hygiene. Molecular detection is more potent and less time consuming than a routine culture and sensitivity. To the best of our knowledge this is the first report on direct molecular detection of bacterial pathogens from urinary tract infected patients in the province of Balochistan. The presence of *bla*_NDM-1_ in different bacterial species and their extensive drug resistance pattern poses a significant clinical health threat. Moreover, direct and early molecular detection of UTI will help the physicians in avoiding irrational prescribing of antibiotics.

### Author’s Contribution:

**GSP, MAA, FA:** conceived, designed and editing of the manuscript. **MD:** Data collection and manuscript writing.

All authors are collectively responsible for accuracy & integrity of the work, especially the principle author (GSP).

## References

[1] Steensberg J, Bartels ED, Bay-Nielsen H, Fanoe E, Hede T (1969). Epidemiology of urinary tract diseases in general practice. Br Med J.

[2] Gupta K, Trautner B (2012). Urinary tract infection. Ann Iintern Med.

[3] Flores-Mireles AL, Walker JN, Caparon M, Hultgren SJ (2015). Urinary tract infections:epidemiology, mechanisms of infection and treatment options. Rev Microbiol.

[4] Freedman AL (2005). Urologic Diseases in America Project. Urologic diseases in North America Project:trends in resource utilization for urinary tract infections in children. J Uurol.

[5] Gales AC, Jones RN, Gordon KA, Sader HS, Wilke WW, Beach ML (2000). SENTRY Study Group Latin America T. Activity and spectrum of 22 antimicrobial agents tested against urinary tract infection pathogens in hospitalized patients in Latin America:Report from the second year of the SENTRY antimicrobial surveillance program (1998). J Antimicrob Chemother.

[6] Rubin RH, Shapiro ED, Andriole VT, Davis RJ, Stamm WE (1992). Evaluation of new anti-infective drugs for the treatment of urinary tract infection. Infect. Dis.

[7] Janda JM, Abbott SL (2007). 16S rRNA gene sequencing for bacterial identification in the diagnostic laboratory:pluses, perils, and pitfalls. J Clin Microbiol.

[8] Mezger A, Gullberg E, Goransson J, Zorzet A, Herthnek D, Tano E (2015). A general method for rapid determination of antibiotic susceptibility and species in bacterial infections. J Clin Microbiol.

[9] Wilson ML, Gaido L (2004). Laboratory diagnosis of urinary tract infections in adult patients. Clin Infect Dis.

[10] Analytical Profile Index API. System S.A Labalmeles Grottes - 38390 montalieu vercieu.

[11] Yan JJ, Wu JJ, Ko WC, Tsai SH, Chuang CL, Wu HM (2004). Plasmid-mediated 16S rRNA methylases conferring high-level aminoglycoside resistance in Escherichia coli and Klebsiella pneumoniae isolates from two Taiwanese hospitals. J Antimicrob Chemother.

[12] CLSI. Performance standards for antimicrobial susceptibility testing, Clinical and Laboratory Standard Institute (2014). CLSI document, Wayne, PA.

[13] Mushtaq S, Irfan S, Sarma JB, Doumith M, Pike R, Pitout J (2011). Phylogenetic diversity of Escherichia coli strains producing NDM-type carbapenemases. J Antimicrob Chemother.

[14] Weisburg WG, Barns SM, Pelletier DA, Lane DJ (1991). 16S ribosomal DNA amplification for phylogenetic study. J Bacteriol.

[15] Manges AR, Johnson JR, Foxman B, O'bryan TT, Fullerton KE, Riley LW (2001). Widespread distribution of urinary tract infections caused by a multidrug-resistant Escherichia coli clonal group. N Engl J Med.

[16] Katouli M (2010). Population structure of gut Escherichia coli and its role in development of extra-intestinal infections. Iran J Microb.

[17] Farooqui BJ, Alam M, Khurshid M (1989). Urinary tract infection. J Pak Med Assoc.

[18] Williams JD, Thomlinson JL, Cole JG, Cope E (1969). Asymptomatic urinary tract infection in gynaecological outpatients. Br Med J.

[19] Vazouras K, Velali K, Tassiou I, Anastasiou-Katsiardani A, Athanasopoulou K, Barbouni A (2019). Treatment and Antimicrobial Resistance in Children with Urinary Tract Infections. J Glob Antimicrob Resist.

[20] Bashir MF, Qazi JI, Ahmad N, Riaz S (2008). Diversity of urinary tract pathogens and drug resistant isolates of Escherichia coli in different age and gender groups of Pakistanis. Trop J Pharm Res.

[21] Rubin RH, Shapiro ED, Andriole VT, Davis RJ, Stamm WE (1992). Evaluation of new anti-infective drugs for the treatment of urinary tract infection. Clin Infect Dis.

[22] Lloyd JC, Hornik CP, Benjamin DK, Clark RH, Routh JC, Smith PB (2016). Incidence of Breakthrough Urinary Tract Infection in Hospitalized Infants Receiving Antibiotic Prophylaxis. Clin. Pediatr.

[23] Chishti AS, Maul EC, Nazario RJ, Bennett JS, Kiessling SG (2010). A guideline for the inpatient care of children with pyelonephritis. Ann Saudi Med.

[24] Poirel L, Revathi G, Bernabeu S, Nordmann P (2011). Detection of NDM-1-producing Klebsiella pneumoniae in Kenya. Antimicrob Agents Chemother.

[25] Deshpande P, Rodrigues C, Shetty A, Kapadia F, Hedge A, Soman R (2010). New Delhi Metallo-beta lactamase (NDM-1) in Enterobacteriaceae:Treatment options with carbapenems compromised. J Assoc Physicians India.

